# Marriage is a Partnership

**Published:** 1888-01

**Authors:** 


### MARRIAGE IS A PARTNERSHIP.

Marriage is still only too often a bargain, but at least, it is no longer an entirely one-sided bargain. It is tending toward the only true ideal of life-long companionship—a partnership on equal terms, with equal give-and-take on both sides. Women no longer feel bound to render that implicit obedience which was considered *de rigueur* in our great grandmother's days, and men no longer universally demand it.

Husbands, moreover, are beginning to learn that their prime duty is not "to look after" their wives. The very sentence is indicative of the most ghastly misapprehension of the whole ideal of matrimony. The general feeling of society condemns a man who lives to rule his wife on the same principles as a Pasha rules his harem.

And indeed, the whole scheme of modern life makes it practically impossible for him to do so. A married woman enjoys, as a rule, complete liberty during the live-long day, and even at night it is frequently impossible for a busy man to escort his wife. Thus everything turns on the relations between the married couple.

If a girl is really in love with the man she marries, she may be trusted with any amount of subsequent freedom. If not, not; and therefore we say that the injudicious and worldly parents who are responsible for the great majority of ill-assorted unions are also responsible for the many evil results which are to be seen in society at this day.

For it is a fact that rows of English girls are as much forced into marriage as the French girl, whose husband is selected while she is yet in in her convent. Not by main force, no ; but by the whole tone of her education ; by the exaggerated fea of being an old maid ; by the obvirous necessity of making way for ayounger sister ; by the persistent scheming of her parents, and by her own longing for emancipation.

For marriage undoubtedly does mean emancipation to most women, and it is precisely those who look forward to it most who are likely to make the worst use of it.—*Pall Mall Gazette.*

